# Comparing the Diagnostic Accuracy of Procalcitonin and C-Reactive Protein in Neonatal Sepsis: A Systematic Review

**DOI:** 10.7759/cureus.19485

**Published:** 2021-11-11

**Authors:** NagaSpurthy Reddy Anugu, Safeera Khan

**Affiliations:** 1 Pediatrics, California Institute of Behavioral Neurosciences & Psychology, Fairfield, USA; 2 Internal Medicine, California Institute of Behavioral Neurosciences & Psychology, Fairfield, USA

**Keywords:** biomarkers, sepsis, neonate, c-reactive protein, procalcitonin

## Abstract

Neonatal sepsis remains a significant diagnostic challenge in newborn care. It has the potential to be disastrous, but precise diagnosis is difficult. No biomarker has yet demonstrated sufficient diagnostic accuracy to rule out sepsis when clinical suspicion exists. As a result, neonates with suspected sepsis are treated with empiric antibiotics. These unnecessary antibiotics promote bacterial antibiotic resistance, raise economic costs, and alter the composition of the gut microbiota. This study aimed to determine the diagnostic accuracy of procalcitonin in the prompt diagnosis of neonatal sepsis.

Articles were systematically screened in PubMed/MEDLINE, PubMed Central (PMC), and ScienceDirect, using keywords and Medical Subject Heading (MeSH) terms to identify the relevant articles. Additionally, one article from the Indian Journal of Applied Research was also used. Inclusion/exclusion criteria were applied post article screening via title and abstracts. Quality appraisal check was done using the Scale for the Assessment of Narrative Review Articles (SANRA) checklist, A Measurement Tool to Assess Systematic Reviews (AMSTAR) checklist, and Newcastle-Ottawa checklist. Six related articles were strictly reviewed.

Procalcitonin is a useful biomarker in the early diagnosis of neonatal sepsis. Because procalcitonin has a better correlation with proven sepsis and is an early biomarker in diagnosing neonatal sepsis, it should be included in the overall sepsis evaluation. Future clinical trials on optimal cut-off levels of procalcitonin with shifting neonatal ages and its use in the post-op setting are needed.

## Introduction and background

Newborn sepsis (NS) is a major cause of neonatal morbidity and mortality, and it has become a serious global public health issue [1,2]. Because the clinical appearance of NS can be confounded with non-infectious conditions, the onset of sepsis might be fast, and the clinical process can swiftly subside. The timely and correct diagnosis of NS is typically challenging in everyday clinical practice. Improving diagnostic testing accuracy may improve outcomes in people with actual sepsis and reduce the indiscriminate administration of antibiotics in those who do not have sepsis [3].

While many impoverished countries still use a substandard approach to diagnose NS, the disease’s non-specific signs and symptoms have made it even more difficult for most modern medical settings to make a precise clinical diagnosis [4].

Microbial cultures can aid in diagnosing serious bacterial infections. However, they frequently produce false-negative results, particularly after maternal antibiotic usage, and may also produce false-positive results due to sample contamination. Furthermore, microbial cultures have a time lag (two-three days) in generating results. As a result, newborns with clinical signs of sepsis or risk factors for serious bacterial infections are typically treated with antibiotics while microbiology testing results are awaited [5]. This eventually leads to antibiotic overuse, resulting in the growth of numerous drug-resistant bacteria in the neonatal intensive care unit (NICU) [6,7]. To prevent microbial resistance from unnecessary empirical treatment and minimize unnecessary hospitalization, a definitive diagnosis based on laboratory testing with greater diagnostic value should be ensured [8]. Biomarkers can aid in the rapid diagnosis of sepsis, the differential diagnosis of non-infectious disorders, and the decision-making process for initial treatment. C-reactive protein (CRP) is an acute-phase protein produced by the liver in response to inflammatory and/or infectious stimuli [9,10]. In the absence of systemic infection, CRP may be elevated in various prenatal circumstances such as fetal distress, stress delivery, and maternal fever [9]. That being the case, its specificity is limited, and it is best used in conjunction with another serum biomarker.

Many writers consider procalcitonin (PCT) to be a promising marker for diagnosing NS among the different molecules studied as biomarkers of sepsis [11]. Despite the fact that PCT is now widely used to diagnose NS in many countries because of its speed and accuracy, questions about its cut-off level for distinguishing NS, appropriate diagnostic values, and the best timing to measure the PCT in NS are still being discussed.

We conducted our systematic review to evaluate the diagnostic value of the test in neonates and to determine the best time to utilize the PCT since determining the best evaluation time can help attending physicians make better clinical judgments, avoid discriminatory PCT testing, and save money in the lab.

## Review

Methods

This systematic review was designed, and its results were reported and principles were adhered to the Preferred Items for Systematic Reviews and Meta-Analysis (PRISMA) 2020 guidelines [12].

Search strategy and data extraction**

We searched PubMed/MEDLINE, PubMed Central (PMC), and ScienceDirect thoroughly to identify full-text relevant published papers. They were meticulously searched using appropriate keywords and Medical Subject Headings (MeSH) terms to find all potentially relevant articles demonstrating the diagnostic accuracy of PCT and CRP in NS. The MeSH strategy used in PubMed was: Procalcitonin OR proinflammatory marker OR ("Procalcitonin"[Mesh]) AND ("Procalcitonin/blood"[Mesh] OR "Procalcitonin/immunology"[Mesh]) AND C-Reactive Protein OR ("C-Reactive Protein/blood"[Mesh] OR "C-Reactive Protein/immunology"[Mesh] OR "C-Reactive Protein/therapeutic use"[Mesh]) AND Neonatal Sepsis OR Neonatal septic shock OR Neonatal pyemia OR Neonatal septic infection OR Neonatal septicemia OR Neonatal toxemia OR Neonatal inflammation OR ("Neonatal Sepsis/blood"[Majr] OR "Neonatal Sepsis/diagnosis"[Majr] OR "Neonatal Sepsis/mortality"[Majr] OR "Neonatal Sepsis/prevention and control"[Majr]). For other databases, the keywords used include procalcitonin, c-reactive protein, and neonatal sepsis.

After retrieving all papers and rigorously checking references to ensure that no potentially relevant publications were overlooked, the titles, abstracts, and subject headings were reviewed for relevance. Primary and secondary outcomes were identified, and the data was extracted by the corresponding authors. Any differences in data extraction were settled through consensus.

Inclusion and exclusion criteria

We included articles over the last five years that were published in English. The neonatal population was the focus of our research. Clinical trials, observational studies, randomized controlled trials, reviews, meta-analysis, and systematic reviews were included. Articles focusing on the adult or geriatric population, unpublished or gray literature, or animal research were omitted.

Risk of bias assessment

We used the following tools to assess the quality of the included studies which is shown in Table 1. Only those articles that satisfied >70% of the checklist quality parameters were included in the review.

**Table 1:** Quality Appraisal Tools

Quality appraisal tools	Articles
Assessing the Methodological Quality of Systematic Reviews (AMSTAR) Checklist	Systematic reviews and meta-analysis
Newcastle-Ottawa checklist	Observational studies
Scale for the Assessment of Narrative Review Articles (SANRA) checklist	Research paper w/out methods section

Results

Study identification and selection

**Figure 1:**
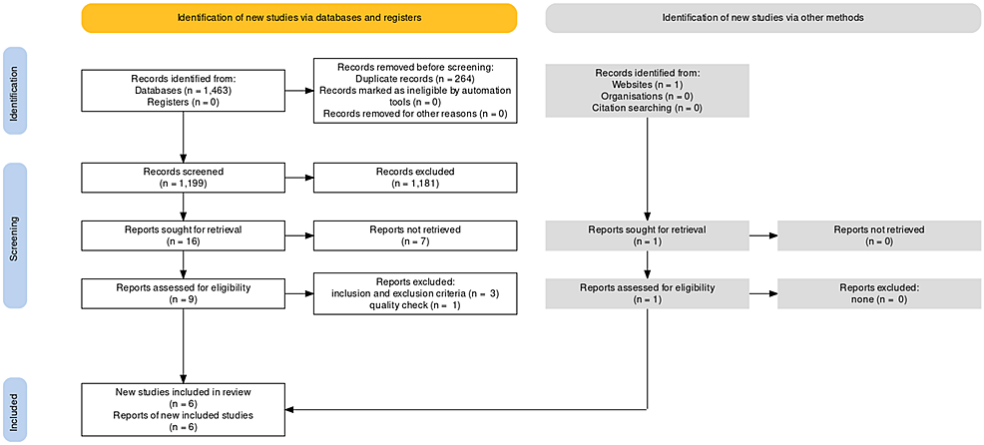
PRISMA Flow Diagram Adapted from Page et al. [12]. PRISMA: Preferred Reporting Items for Systematic Review and Meta-Analyses

A total of 1464 articles were identified using the various search strategies employed. Out of 1464 articles, 1399 articles were from PubMed, 64 studies from ScienceDirect, and one article was obtained via reference perusal; 1200 articles remained after removing 264 duplicate articles. We then filtered the remaining articles based on the relevance of the title and contents of their respective abstracts to our ongoing research. Out of which, 1181 articles were discarded due to irrelevance. Hence, 19 articles were left, and we checked for availability of full texts, out of which seven articles were removed due to unavailability of the full text. Out of the remaining articles, nine were found eligible based on the eligibility criteria. Six articles were finalized after the quality assessment-Two observational studies, one traditional review, one meta-analysis, and two systematic reviews. A complete Preferred Reporting Items for Systematic Reviews and Meta-Analyses (PRISMA) flow diagram is shown below in Figure 1.

The brief description of the studies included in the review is shown in Table 2.

**Table 2:** Summary of Studies Included in Systematic Review CRP: C-reactive protein; PCT: procalcitonin; NS: neonatal sepsis

Study and year of publication	Study type	Purpose of study	Result/conclusion
Rashwan et al., 2019 [13]	Cross-sectional study	To determine the validity of biomarkers in screening for NS	CRP—more valuable in late-onset NS. PCT, presepsin, and hs-CRP, when used together, were early diagnostic markers for NS
Eschborn and Weitkamp, 2019 [14]	Systematic review	Review of kinetics and performance of PCT and CRP for diagnosis of NS	PCT and CRP perform better when measured serially to be used along with other clinical and laboratory data for initiation/stoppage of antibiotics
Ruan et al., 2018 [15]	Systematic review and meta-analysis	To evaluate the accuracy of diagnosis of NS using PCT and CRP combined or presepsin alone	PCT and CRP together improves the accuracy of the diagnosis of NS
Sharma et al., 2018 [16]	Literature review	Review of biomarkers for diagnosis of NS	CRP, PCT—most commonly used as sepsis markers. There is still a need to find an ideal biomarker
Liu et al., 2019 [17]	Meta-analysis	To assess the accuracy of CRP in neonatal septicemia	CRP can be used in detecting NS. But serum PCT has high specificity and sensitivity in diagnosing early NS
Thota et al., 2016 [18]	Observational study	To evaluate the role of PCT in the diagnosis of NS	PCT has a better correlation with confirmed sepsis. Therefore, it should be included in a full sepsis evaluation

Discussion

Early diagnosis and treatment are the key elements of improving survival in sepsis syndrome. The ability to appropriately identify newborns with culture-negative sepsis who require antibiotic therapy is a significant advantage of employing biomarkers in screening for NS.

The lack of an acceptable reference standard is the first difficulty in evaluating a diagnostic strategy for NS. An ideal marker is the one that has both high sensitivity and high specificity. High sensitivity is to avoid missing even a single case of NS while high specificity is to avoid unnecessary antibiotic exposure. There is still a need to establish the “ideal” biomarker because none of the ones commonly used in clinical practice has 100% sensitivity and specificity.

The laboratory investigations used for diagnosing neonatal sepsis are included in Table 3.

**Table 3:** Laboratory Tests for Diagnosis of Neonatal Sepsis ESR: erythrocyte sedimentation rate; C5a: complement component 5a; C5L2: complement 5a-like receptor; IL-1: interleukin-1; IL-6: interleukin-6; IL-8: interleukin-8; IL-1ra: interleukin-1 receptor antagonist; IL-2rs: interleukin-2 receptor subunits; IL-10: interleukin-10; RANTES: regulated on Activation, Normal T cell Expressed and Secreted; TNF-α: tumor necrosis factor-α; IFN-γ: interferon-γ; G-CSF: granulocyte colony-stimulating factor; CSF1: colony stimulating factor 1; SCF: stem cell factor; MIP1-a: macrophage inflammatory protein-1 alpha; sCD14: soluble cluster of differentiation 14; sICAM-1: soluble intercellular adhesion molecule-1; CD11b: cluster of differentiation molecule 11b; CD64: cluster of differentiation 64; CD69: cluster of differentiation 69; CD25: cluster of differentiation 25; CD19: cluster of differentiation 19; CD33: cluster of differentiation 33

Specific laboratory tests	Hematologic investigations	Biochemical investigations	Cytokines and receptors
Blood, cerebrospinal fluid, and urine culture	White blood cell counts	C-reactive protein, procalcitonin	IL-1, IL-6, IL-8, IL-1ra, IL-2rs
Direct visualization of bacteria (Gram stain)	Total and differential, platelet counts	ESR, serum amyloid	IL-10, RANTES, TNF-α, IFN-γ
Detection of bacterial antigens		other phase reactants: haptoglobin, lactoferrin, neopterin, inter-inhibitor proteins, lipopolysaccharide-binding protein, C5a, C5L2, immunoglobulins	G-CSF, CSF1, SCF, MIP1-a
Polymerase chain reaction (amplification of bacterial DNA)			sCD14, sICAM-1, CD11b, CD64, CD69, CD25, CD19, CD33

The NS evaluation test is important because infection implies a very serious threat to neonates. CRP is the most commonly used biomarker for identifying NS in the NICU, owing to its low cost, ability to be performed at all centers, and easy availability of test results. Several studies, however, suggest that PCT is a more sensitive and specific marker in the pediatric and adult populations [19].

This systematic review attempts to comprehensively assess the diagnostic value of PCT level as an early marker to detect NS. While few studies directly compare PCT and CRP, there is a significant body of literature that offers performance measures for individual markers.

Diagnostic Accuracy of C-Reactive Protein

A meta-analysis of the accuracy of the CRP test for neonatal septicemia found an overall sensitivity of 71% and specificity of 86% in the diagnosis of NS [17]. Serum CRP levels are quite low under normal conditions; when a person is infected with bacteria, the body releases endogenous neurotransmitters to excite liver cells via white blood cells (WBCs) and other inflammatory cells. CRP synthesis occurs within 4-6 h and peaks at 36-50 h. Therefore, the inflammatory process typically begins 6-12 h following CRP detection [14,17]. NS can lead to elevated CRP. The increase in CRP serum concentration is comparatively slow during the first 24-48 h of infection, which may reduce the test’s sensitivity. Furthermore, an increase in CRP levels in non-infected clinical circumstances like meconium aspiration and prolonged rupture of membranes are thought to impact the test’s specificity. CRP not only has lesser sensitivity and specificity for sepsis diagnosis than PCT but it also has a slower descent pattern in comparison to PCT [17].

Diagnostic Accuracy of Procalcitonin

As already discussed, the CRP is the most important component of the sepsis screen. But a study conducted in 2016 [18] stated that PCT has a better profile with a quick rise following exposure to bacterial endotoxin, allowing for a faster diagnosis. Because blood culture is considered the gold-standard test for sepsis, it is logical to compare the efficacy of CRP and PCT to blood culture results. According to the findings of this study, PCT is more sensitive than CRP in detecting sepsis early. Thota et al. concluded that PCT is a sensitive, independent, and useful biomarker compared to CRP in early diagnosis of neonatal sepsis, i.e., PCT showing better sensitivity, specificity, positive predictive value (PPV), and negative predictive value (NPV) than CRP. It may also be a better differentiator between viral and bacterial infection, albeit this must be proven in newborns [18].

Supporting the above statement, according to Sharma et al., the rise of CRP after the onset of sepsis is comparatively slower and has low sensitivity early in sepsis [16]. PCT has been linked to immunomodulation and vascular response in systemic inflammatory response syndrome (SIRS), particularly in patients with systemic bacterial infection. The PCT level rises rapidly within 2-4 h following bacterial endotoxin exposure, peaks at 6-8 h, and remains elevated for the next 24 h [14]. PCT has a half-life of 24-30 h. Another advantage of PCT is that its serum concentrations remain high compared to other sepsis biomarkers such as tumor necrosis factor-alpha (TNFa) and IL-6, making PCT more useful in predicting infection severity and responsiveness to treatment [16].

A systematic review published in 2018 concluded that PCT is more sensitive than CRP but that doing the two tests together would result in greater sensitivity and would be more useful in the detection of sepsis [15]. Furthermore, excluding the diagnosis of sepsis is important so that the number of neonates treated with antibiotics can be reduced, hospital stays can be reduced, selection pressure for resistant strains appears to be lower, and medical and economic benefits may offset the financial costs of measuring PCT.

It was found in our review that five out of six studies evaluated the role of PCT in diagnosing NS. All five studies showed a positive correlation between PCT and the diagnosis of sepsis. All five studies compared PCT to CRP and showed PCT has higher sensitivity and accuracy in diagnosing sepsis. Compared to CRP, the quick rise in PCT with the start of bacterial sepsis makes it an excellent marker for early detection of NS. We must also keep in mind that not all patients with infections are septic. However, studies have shown that patients with sepsis have considerably greater PCT levels than those with an isolated infection, allowing us to identify vulnerable patients who require more comprehensive treatment [13], i.e., PCT levels are highly elevated in all neonatal cases of sepsis, and their level correlates with the severity of the infection [13,20].

The use of biomarkers is one area that continues to hold promise for the early identification of patients with sepsis. Traditional individual markers of sepsis, like the entire leukocyte count, neutrophil count, and CRP, lack the specificity to permit them to discriminate between those patients with an inflammatory response to trauma or surgery, for instance, and people with a new infection. In this sense, PCT has been shown to possess the simplest accuracy in spotting patients with invasive bacterial infections [21]. PCT production can also be induced by non-infectious causes of systemic inflammation, such as shock, trauma, surgery, burn injury, etc. However, it is observed that the rise of PCT levels in non-infectious causes is not as significant as the elevation of PCT levels in sepsis [16,22,23].

PCT has the benefit of rising more quickly than CRP. However, its application has been limited in comparison to the CRP due to extremely rapid fluctuations in baseline levels after birth and the need for multiple different cut-off values with shifting neonatal age. The therapeutic utility of employing NS biomarkers is still being debated. As a result, more research is needed to determine the best cut-off levels for PCT in neonates so that it can be used consistently in clinical trials. Biomarker kinetics should be considered for sample timing in future studies. More research is needed to determine whether PCT is beneficial for predicting the most severe consequences of sepsis in the critical care setting. The evidence for its use in the postoperative setting also needs to be carefully evaluated.

This systematic review showed that determining PCT levels in blood was linked to a decrease in the number of diagnostic tests conducted and patients treated with antibiotics. It has also enabled the early identification of infected patients and, by distinguishing between newborns at higher and lower risk, has allowed for a reduction in the time the latter spend under monitoring, which has favored mother-child bonding and the maintenance of breastfeeding. Implementing a strategy in clinical practice that can distinguish patients at risk of infection that can be managed conservatively, as most at-risk newborns will not develop sepsis, might benefit. However, more research is needed to back this up.

Limitations

The quality of the primary studies varied due to confounding variables. Furthermore, the individual patient differences, diagnostic criteria for NS, methods for detecting samples, laboratory testing levels, and instruments used can be the reason behind the variability in the results among studies. Different studies utilized different statistical approaches to measure outcomes, making it difficult to integrate results, which is a limitation of this systematic review.

## Conclusions

This systematic review was conducted to determine whether PCT is a more accurate early diagnostic marker in NS than CRP. PCT seems to be one of the most promising biomarkers of sepsis among the several compounds being researched. The rise of PCT after the onset of sepsis is comparatively faster. It has more sensitivity early in sepsis than CRP, thereby ensuring a definitive diagnosis, reducing hospital stay, antibiotic overuse, and microbial resistance. Moreover, it can also predict the severity of infection and responsiveness to treatment based on its serum concentration. Although this needs to be proven in newborns, PCT may be a better differentiator between viral and bacterial infection.

Larger, high-quality studies are needed to further our scientific understanding of the function of PCT in the diagnosis of NS. More specifically, a future recommendation in this area is conducting more clinical trials to check the cut-off levels of PCT with shifting neonatal age considering factors affecting it and its use in post-op settings.
